# Supplementary material for: The genome sequence of the sweet violet, Viola odorata L. (Malpighiales: Violaceae)
Source: Wellcome Open Res. 2026 Mar 18;11:177. [Version 1] doi: 10.12688/wellcomeopenres.26151.1 (PMC13058574; doi:10.12688/wellcomeopenres.26151.1)
Supplement: Peer review history

# Reviewer report: Reviewer response for version 1

**DOI:** 10.21956/wellcomeopenres.28798.r151785

**Published:** 2026-06-02

**Authors:** Zoé Postel

**Affiliations:** 1 Department of Ecology, Environment and Plant sciences, Stockholm University, Stockholm, Sweden

This report by Christenhusz et al. describes the genome assembly of *Viola odorata* (Malpighiales) based on an individual sampled in Surrey, UK. The motivation, methodology, and assessment of assembly quality are clearly presented and easy to follow. Overall, the manuscript is well written, and the level of detail provided should allow future users to readily assess the quality and usability of the genome resource. The assembly itself appears to be of high structural quality, with excellent BUSCO completeness and a very high proportion of the assembly anchored to chromosome-level scaffolds. This represents a valuable resource for future research in plant biology and genomics!

 Additional details on the annotation process would be beneficial, although the authors appropriately refer to established protocols.

 Overall, this work provides a useful and well-documented genomic resource.

 Some more specific comments can be find below:

- p. 5 - In the Methods (genome assembly section), k-mer counts were performed on filtered reads. It would be helpful to clarify how these reads were filtered and which criteria were applied.
- p. 5 - For mitochondrial and chloroplast genome assembly, the authors used two different tools (MitoHiFi and OATK). Could the authors clarify the rationale for this choice, given that OATK can be applied to both organellar genomes?
- Figure 2 would benefit from additional explanation to help a broader audience interpret the different peaks.
- Figure 6 : a bit more help would be appreciated for interpretation from the reader
- p. 11 - The high proportion of duplicated BUSCOs would merit further discussion. Given the reported level of heterozygosity (7.22%) and very low k-mer completeness for the alternate assembly (1.3%), this may reflect incomplete collapsing of heterozygous regions in the primary assembly.
- p. 11 - Among the 34,902 protein-coding genes, it would be informative to specify how many are of mitochondrial or chloroplast origin.

Are sufficient details of methods and materials provided to allow replication by others?

Yes

Is the rationale for creating the dataset(s) clearly described?

Yes

Are the datasets clearly presented in a useable and accessible format?

Yes

Are the protocols appropriate and is the work technically sound?

Yes

Reviewer Expertise:

Plant geneticist and population genomics, nuclear and organellar genomes

I confirm that I have read this submission and believe that I have an appropriate level of expertise to confirm that it is of an acceptable scientific standard.

# Reviewer report: Reviewer response for version 1

**DOI:** 10.21956/wellcomeopenres.28798.r151135

**Published:** 2026-04-07

**Authors:** Stalin Nithaniyal

**Affiliations:** 1 Botanical Survey of India, Western Regional Centre, Pune, Maharashtra, India

Christenhusz and co-authors investigated genome sequence of sweet violet ( *Viola odorata*) using Illumina NovaSeq 6000 as a part of Darwin Tree of Life project. This timely study focused on the notable plant *V. odorata*, sampled from Richmond, Surrey, United Kingdom, which is highly regarded for its cultural, horticultural, and medicinal value. The research was well-executed, utilizing a robust methodology covering DNA extraction, library preparation, genome assembly, and curation. The genome assembly represents a high-quality reference data, indicated by its high contiguity (99.9% chromosomal scaffolding) and high accuracy (QV 59.0). Furthermore, the EBP 6.C.Q59 metric demonstrates that it meets the highest international standards for reference-quality genomes. This assembly provides a strong foundation for evolutionary studies, boasting 99.2% gene-space completeness and a comprehensive Ensembl annotation of 34,902 protein-coding genes from mitochondrial, and plastid genomes. While the high gene duplication rate (85.9%) in the BUSCO analysis suggests a need for careful interpretation to distinguish between biological paralogs and potential assembly redundancies, the 99.2% overall BUSCO score confirms that the assembly captures nearly the entire expected gene set. Overall, the work is well-presented, and the genomic data will serve as a valuable resource for botanists and plant geneticists by facilitating future comparative studies.

 Major outcomes of the study are,

 1. Achieving 99.9% of the 698.62 Mb assembly and scaffolded into 10 chromosomal pseudomolecules is a significant result and provide near-complete physical map of the genome.

 2. High QV of 59.0 demonstrates quality base-call accuracy required for the reference genomes

 3. Successful assembly of mitochondrial (482.11 kb) and plastid (158.28 kb) genomes

 4. Ensembl Annotation provides well-curated set of 34,902 protein-coding genes which act as valuable resource to the research community

 Some minor comments includes,

 1. Clarify the reason for high duplication rate in BUSCO analysis (85.9%) and selection of eukaryota_odb10 reference set instead of plant reference

 2. A ratio of 1.2 transcripts per gene is considered low in the context of plant transcriptomics

 3. Breakdown of 14 061 non-coding genes may be provided.

Are sufficient details of methods and materials provided to allow replication by others?

Yes

Is the rationale for creating the dataset(s) clearly described?

Yes

Are the datasets clearly presented in a useable and accessible format?

Yes

Are the protocols appropriate and is the work technically sound?

Yes

Reviewer Expertise:

Molecular Biology, Plant Systematics, Ethnobotany

I confirm that I have read this submission and believe that I have an appropriate level of expertise to confirm that it is of an acceptable scientific standard.

# Reviewer report: Reviewer response for version 1

**DOI:** 10.21956/wellcomeopenres.28798.r151782

**Published:** 2026-04-03

**Authors:** Bruno Contreras-Moreira

**Affiliations:** 1 Estacion Experimental de Aula Dei-CSIC (Ringgold ID: 54439), Zaragoza, Aragon, Spain

The manuscript by Christenhusz et al describes their work on the assembly and annotation of the genome of dicot Viola odorata out of DNA extracted from a plant in Surrey. I find that the methods describe with sufficient precision all the steps from plant identification in a public park to the preparation of DNA libraries, assembly and QC. Probably the part that is explained with least detail is the gene annotation protocol, despite the info available externally. All software version/releases are also listed on Table 5.

 Regarding the results, the paper reports efficiently what most readers will want to know about an assembly, its quality, and its annotation, linking back to the data sources and INSDC. In addition, the genome is made available at the Ensembl browser so that anyone can use it.

 Here are some comments in case the authors want to address them:

 Figure 2 legend: Please help readers understand the figure; as it stands some people won't be able to follow what the peaks are.

 p8: Please provide details of the singularity container for reproducibility.

 p11 "Assembly quality metrics": 

 a) I see the primary hifiasm assembly is reported; did the authors ever try a hap1/hap2 assembly or that pathway is deemed too complidated?

 b) why is the BUSCO completeness reported here relate to lineage eukaryota_odb10 instead of eudicots_odb1o (Figure 5)? Please explain to avoid confusion.

 p11 "Genome annotation report": did the gene annotation of this genome used any RNAseq data from related species or was instead based on de novo or protein-based gene calling?

Are sufficient details of methods and materials provided to allow replication by others?

Partly

Is the rationale for creating the dataset(s) clearly described?

Yes

Are the datasets clearly presented in a useable and accessible format?

Yes

Are the protocols appropriate and is the work technically sound?

Yes

Reviewer Expertise:

Bioinformatics, plant genomics

I confirm that I have read this submission and believe that I have an appropriate level of expertise to confirm that it is of an acceptable scientific standard.
